# Prognostic understanding interventions in patients with advanced cancer: a systematic review

**DOI:** 10.3389/fpsyg.2026.1824994

**Published:** 2026-06-03

**Authors:** Li Dong, Liande Tao, Yulin Wang, Min Shi, Changxia Cheng, Li Li

**Affiliations:** Department of Nursing, The Second People's Hospital of Yibin, Yibin, China

**Keywords:** advanced cancer, intervention, patients, prognostic understanding, systematic review

## Abstract

**Background:**

Most patients with advanced cancer have an inadequate understanding of their prognosis, and there are few interventions aimed at enhancing their prognostic understanding, with the effectiveness of these interventions remaining controversial.

**Aim:**

The purpose of this systematic review is to identify interventions that have been effective at improving prognostic understanding in patients with advanced cancer.

**Methods:**

A systematic review was conducted in accordance with PRISMA guidelines. All relevant peer-reviewed articles published from 1990 and February 2025 were identified through an electronic search of four databases: Embase, Scopus, Pubmed, and Web of Science. Risk of bias was independently assessed by two reviewers using the Cochrane Risk of Bias Assessment Tool (CRBT) and Cochrane Risk of Bias Assessment Tool for Non-randomized Studies (RoBANS). Following this, a narrative synthesis of the findings was completed. Publications reporting on interventions delivered to patients with advanced cancer that included quantitative data on prognostic understanding were systematically reviewed.

**Results:**

Thirteen unique intervention studies involving 1,780 cancer patients were identified. Interventions tested included decision aids (DAs), prognostic discussions, advance care planning (ACP), and palliative care. Seven studies utilized a randomized controlled design. Five of these investigated DAs-based interventions and four examined prognostic discussions interventions, with the greatest empirical support found for these intervention types. However, most interventions fail to improve the prognostic understanding of patients with advanced cancer.

**Conclusion:**

Further research is needed to investigate the effects of interventions on prognostic understanding among patients with advanced cancers, using randomized controlled designs and adequately powered samples. This would enable the implementation of evidence-based recommendations for the most appropriate interventions in clinical practice.

## Background

Among patients with advanced cancer, prognostic understanding is considered essential for optimal, value-concordant, end-of-life planning and treatment decision-making (Chichua et al., 2023). Currently, there is no unified concept of prognostic understanding. Most studies define prognostic understanding as the awareness of metastatic or advanced disease, including the terminal nature of the disease, life expectancy, and incurability, which serves as the foundation for engaging patients in end-of-life discussions (Weeks et al., 1998; Applebaum et al., 2014; Diamond et al., 2014; Chen et al., 2017; Vlckova et al., 2020). In clinical practice, clinicians seldom initiate discussions with patients regarding realistic prognoses and treatment goals. Consequently, it often proves challenging for them to comprehensively address patients’ needs for prognostic information when such inquiries arise. Although most patients wish to receive comprehensive prognostic information, physicians encounter difficulties in disclosing this information. Their concerns arise from the potential to evoke negative emotions in patients, the inherent challenges in accurately estimating survival times, and the inconsistencies in these estimations (Gordon and Daugherty, 2003; Cheon et al., 2016; Stone et al., 2023).

Most patients with advanced cancer reported an overly optimistic assessment of the likelihood of a cure compared to their oncologists (Mathews et al., 2024). In addition, a small number of patients have misconceptions about the prognosis. Even if they recognize that cancers is incurable, they still believe that the goal of chemotherapy is to eliminate all of their cancers (Temel et al., 2011). It is noteworthy that the chance of chemotherapy curing metastatic cancer patients is very low (Schiller et al., 2002; Kris et al., 2003; Sandler et al., 2006).

Due to a lack of understanding about prognosis among patients with advanced cancer, they are unable to make informed choices regarding end-of-life care. Patients with advanced cancer with an accurate understanding of prognosis were more willing to accept less aggressive care at the end of life, prefer to choose home as the place of death, and were more likely to complete do-not-resuscitate orders (Trice and Prigerson, 2009; Waite et al., 2013; Enzinger et al., 2015; van der Velden et al., 2020). The understanding of prognosis in patients with advanced cancer is associated with various factors, including male gender, low education level, the communication of prognosis by physicians, and the patient’s readiness to receive bad news (Costello, 2000; Butow et al., 2002; Back et al., 2005; Yanwei et al., 2017).

There are limited studies published on interventions targeting prognostic understanding. The results of these studies vary widely. Such as, some research indicates that prognostic communication interventions for patients with advanced cancer, which convey critical aspects of end-of-life decision-making, can enhance patients’ understanding of their prognosis (Step et al., 2019; Prigerson et al., 2023). Conversely, other studies have reported no significant effects (Nakano et al., 2018; Shen et al., 2024).

George et al. (2022) have conducted a review of interventions on prognostic awareness, but the included population was not limited to patients with advanced cancer, it also encompassed those with chronic heart failure, and it did not clearly indicate which intervention component or type was more effective. This systematic review aims to systematically sort out the interventions designed to enhance the prognostic understanding of patients with advanced cancer. Unlike previous review, this systematic review offers an updated synthesis that includes the most recent studies published between 1990 and 2025. It compares characteristics such as cancer types, the quality of doctor-patient communication, and patients’ sociocultural backgrounds to identify factors that may influence the effectiveness of various interventions. Additionally, it provides recommendations for future research aimed at improving prognostic understanding within this population.

## Methods

This systematic review conforms to the Preferred Reporting Items for Systematic Reviews and Meta-Analyses (PRISMA) statement. A systematic review of published research was conducted to determine the effectiveness of interventions which improve prognostic understanding in patients with advanced cancer. We searched Embase, Scopus, Pubmed, and Web of Science from 1990 to February 12th, 2025, using a pre-defined search strategy developed in collaboration with experienced librarians. The search strategy (see Supplementary file 1) incorporated terms related to patients with advanced cancer, prognostic understanding, and various interventions, tailored specifically for each database. The starting point of our search strategy was to include a broad range of terms relating to prognostic understanding, in order to retrieve all relevant papers. For instance, terms such as ‘prognostic awareness’, ‘prognosis’, ‘prognoses’, ‘life expectancy’, and ‘illness perception’ were employed due to their similar meanings to prognostic understanding.

### Eligibility criteria

The Participant, Intervention, Comparators and Outcomes (PICO) framework was applied to determine the eligibility criteria (Table 1). Due to the limited use of randomized controlled trials (RCTs) targeting prognostic understanding among patients with advanced cancer, pre–post methods with quantitative reporting were also included. Exclusion criteria included articles, books, or book chapters written in languages other than English, articles reporting on populations other than cancer patients, articles reporting only qualitative results, and literature/systematic reviews.

**Table 1:** PICO framework.

PICO	Description
Participant	Patients (>18 years of age) with advanced (incurable) cancer
Intervention	Any intervention which has been designed to improve prognostic understanding
Comparators	Usual care
Outcomes	All outcome measures used for prognostic understanding or related themes (such as patients’ awareness of the advanced nature of cancer, life expectancy, and incurability)

Two authors (LD and LL) independently conducted a database search and screened the titles and abstracts of potentially relevant sources based on the established inclusion and exclusion criteria. The reference management software Endnote (version X9.0) was used to remove the duplicate records. The full texts of the relevant articles were then reviewed by both authors to identify studies suitable for inclusion in the review. Additionally, supplementary methods such as gray literature search, manual search, and reference citation search were employed to identify further relevant studies. Ultimately, the authors discussed their findings and reached a consensus, with any disagreements being resolved through consultation with a third author (MS).

### Data extraction and quality appraisal

Data regarding study characteristics, participant demographics, outcome definitions, and intervention results were independently extracted from the eligible articles by two authors (LD and LL) using a structured extraction form in Microsoft Excel. All entries were cross-checked for accuracy. In addition, when some data is missing, we will contact the author via email for information. Two authors independently assessed the risk of bias and resolved discrepancies through discussion. When discrepancies could not be resolved, they consulted a third author (MS).

The risk of bias of Randomized controlled trials (RCTs) were assessed using the Cochrane Risk of Bias Tool (CRBT; Higgins et al., 2011). The risk of bias of Non-RCT studies were evaluated using the Cochrane Risk of Bias Assessment Tool for Non-randomized Studies (RoBANS; Kim et al., 2013). The following domains of each study were assessed: random sequence generation, allocation concealment, blinding of participants and personnel, incomplete outcome data, selective reporting or other. Based on these domains, the overall risk of bias for each included study was evaluated.

### Analysis

Due to heterogeneity in participants, interventions, and outcomes, overall effect sizes could not be calculated.

## Results

### Search outcomes

**Figure 1:**
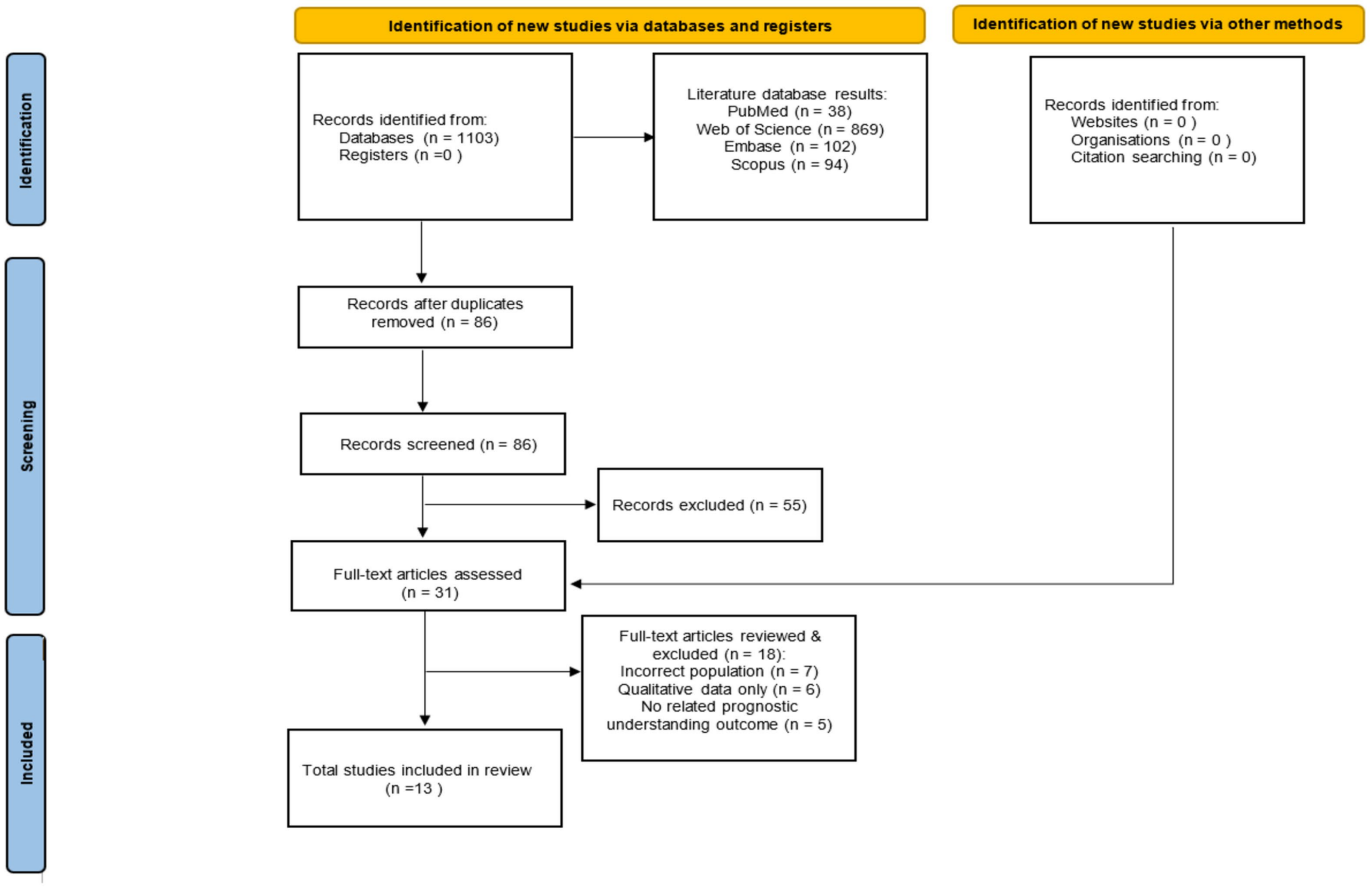
PRISMA flow diagram at each stage of the selection process.

A total of 1,103 studies were identified through electronic database searching. After screening titles and abstracts and removing duplicate literature, 13 articles met our inclusion criteria (Figure 1).

### Study characteristics

The 13 studies involving 1,780 patients with advanced cancer. All studies were published between 2011 and 2024, and most studies were published in the past 5 years. Table 2 summarizes the general characteristics of the included articles. Nine of the 13 articles were conducted in the US, and the others were conducted in Saudi Arabia, Japan, China, Australia and Canada. Of the articles reviewed, seven were randomized controlled trials, while six were observational studies (including one *post-hoc* analysis associated with one of the seven RCTs). These studies are heterogeneous: they involve different types of cancer, using different methodologies, and have variable inclusion criteria.

**Table 2:** Details of included articles.

Author & year	Study type	Country	Total size	Participants	Interventionist/s	Brief description of intervention	Definitions of prognostic understanding	Prognostic understanding outcomes	Risk of bias
Leighl et al. (2011)	RCT	Australia and Canada	207	Patients with incurable metastatic colorectal cancer	Oncologists	Patients in the intervention group were provided with a take-home version of the Decision Aid following the initial consultation. Oncologists were trained to use Decision Aid during consultations and patients were instructed to return after the initial consultation to make final treatment decisions. Decision Aid provides patients with more information about treatment options and goals of palliative treatment with or without chemotherapy in the form of manuals with audiotape or compact disk	Understand the impact of chemotherapy and adverse effects; understand about survival outcomes	Prognostic understanding was significantly improved in the intervention group compared to the control group	Low
Temel et al. (2011)	RCT	USA	151	Patients with metastatic non–small-cell lung cancer	Palliative care team (including palliative care physicians and advanced-practice nurses)	Patients in the intervention group received early palliative care combined with standard oncology therapy, the palliative care clinicians focus on patient understanding and education of disease, symptom management, treatment decisions, coping with a life-threatening illness for patients and families, and develop advance care planning	Perceptions of prognosis and goals of therapy	Patients in the intervention group were more likely to retain or develop an accurate assessment of their prognosis than those in the control group, however, there was no significant difference between the two groups in their perceptions of treatment goals	High
Nakano et al. (2018)	Pretest– posttest	Japan	20	Patients with advanced non–small-cell lung cancer	Physician and palliative care team (psycho-oncologist and oncology nurse)	Providing patients with a three-page information aid pamphlet, the first and second pages of the pamphlet containing chemotherapy-related information on diagnosis, treatment goals, toxicity, regimens and schedules, and treatment options, the third page containing survival data from the Decision Aid for first-line cytotoxic chemotherapy	Perception of cure and life expectancy	No difference between groups in prognostic understanding	High
Step et al. (2019)	RCT	USA	28	Patients with advanced breast cancer	Team (including medical oncologist, nurse practitioner, and oncology social workers)	The T-PAT intervention is a 60-min, patient education appointment. Physicians help patients clarify prognosis and treatment goals, nurses help patients establish supportive care networks, and social workers assess patients ‘current health status and long-term planning goals	Strength of Belief in Curability: perception of cure	A higher proportion of patients in the intervention group had a more accurate understanding of the curability of the disease	High
AlSagheir et al. (2020)	RCT	Saudi Arabia	92	Patients with metastatic colorectal cancer	Nurse	Decision Aid convey information to patients about palliative care goals, treatment options, treatment administration, benefits and risks via booklet and audio recording	Awareness of toxicity caused by chemotherapy; awareness of the purpose of chemotherapy; awareness of the consequences of not receiving chemotherapy; awareness of the disease is incurable	No difference between groups in prognostic understanding	High
Lippe et al. (2020)	Pre-post and qualitative research	USA	62	Patients with incurable cancer	Oncologists	Oncologists use the Communicating Oncologic Prognosis with Empathy communication guidelines to communicate with patients, focusing on patient outcomes, patient needs and goals	The Prognosis and Treatment Perception Questionnaire: chance of cure; information about prognosis; goals of care; treatment information preferences; and satisfaction with quality of information provided	Compared to before the intervention, patients had a more accurate understanding of the prognosis after the intervention	Low
Chen et al. (2020)	Secondary analysis of a RCT	China	460	Patients with advanced cancer	Nurses	Assess patients’readiness for ACP, facilitate prognostic communication and end-of-life care discussions among patients, family caregivers, and physicians, and use booklet and video as educational aid to facilitate patient understanding of ACP and life-sustaining treatments at end-of-life	Awareness of the possibility of a cure	Accurate prognostic understanding was higher in the intervention group than in the control group	High
Nipp et al. (2020)	Pilot RCT	USA	62	Patients with incurable gastrointestinal and lung cancer	Geriatricians	Patients completed a brief geriatric screening tool prior to their initial visit, and geriatricians discussed and addressed the patient’s physical and psychological symptoms, comorbid status and multiple medications, cognitive issues, availability of social support, functional impairment, and use of coping strategies	Threatening perception of illness, assessment using the Brief Illness Perceptions Questionnaire	No difference between groups in prognostic understanding	Low
Enzinger et al. (2020)	RCT	USA	186	Patients with advanced colorectal and pancreatic cancer	Oncologists	Patients were given appropriate educational booklets and videos containing information on infusion logistics, treatment objectives, treatment benefits, side effects, alternatives, and sample patient experiences	Understanding of chemotherapy benefits, goals, and side effects	No difference between groups in patients’ expectations for cure from palliative chemotherapy, the intervention group demonstrated a significantly higher correct understanding of adverse reactions to chemotherapy compared to the control group, but there was no statistical difference	High
Enzinger et al. (2021)	Secondary analysis of a RCT	USA	186	Patients with advanced colorectal and pancreatic cancer	Oncologists	Patients were given appropriate educational booklets and videos containing information on infusion logistics, treatment objectives, treatment benefits, side effects, alternatives, sample patient experiences, life expectancy	Understanding of life expectancy	In the overall study population, there was no difference in the estimation of life expectancy between the intervention group and the control group. However, in the pancreatic cancer subgroup, patients in the intervention group provided more accurate estimations of life expectancy, compared to the control group	High
Sigler et al. (2022)	Secondary analysis of a RCT	USA	457	Patients with advanced Cancer	Nurses	CONNECT is a primary palliative care intervention led by oncology nurses, nurses first build rapport with patients, address patients’ symptom needs, and identify surrogate decision makers, then focus on patient perceptions of disease, ongoing symptom management, and advance care planning	Perceptions of life expectancy, treatment intent, and terminal illness acknowledgment	No difference between groups in prognostic understanding	High
Prigerson et al. (2023)	Pilot RCT	USA	33	Patients with advanced cancer	Oncologists	Using simple words and phrases to communicate with patients about the condition, life expectancy, medical recommendation, and finally ask if the patients understand the communication	Perception of cure, disease stage, and life expectancy	No difference between groups in prognostic understanding	High
Shen et al. (2024)	Pretest– posttest	USA	*N* = 22 patients and *N* = 22 caregivers	Patients with advanced cancer; caregivers	Social workers	The Talking About Cancer intervention centered on distress management strategies, communication skills, prognostic information and advance care planning information, and plan for managing future difficult conversations	Perception of the nature of disease; life expectancy estimate	No difference between groups in prognostic understanding	High

RCT, Randomized Controlled Trial; T-PAT, Team based prognosis and treatment goal discussion; ACP, Advance Care Planning; CONNECT, Care management by Oncology Nurses to address supportive care needs.

Three studies looked at interventions delivered by teams consisting of physicians, nurses, and/or social workers (Temel et al., 2011; Nakano et al., 2018; Step et al., 2019), one study looked at interventions provided by social workers (Shen et al., 2024), five studies looked at interventions delivered by oncologists (Leighl et al., 2011; Enzinger et al., 2020; Lippe et al., 2020; Enzinger et al., 2021; Prigerson et al., 2023), three studies looked at interventions delivered by nurses (AlSagheir et al., 2020; Chen et al., 2020; Sigler et al., 2022) and one study looked at interventions delivered by geriatricians (Nipp et al., 2020). However, several studies (AlSagheir et al., 2020; Enzinger et al., 2020; Enzinger et al., 2021) have primarily relied on providing educational materials—such as booklets, videos, or audio recording—directly to patients, the direct involvement of the interventionists is relatively low.

Five studies focused on specific cancer patients: colorectal cancer patients (*N* = 2; Leighl et al., 2011; AlSagheir et al., 2020), breast cancer patients (*N* = 1; Step et al., 2019), and lung cancer patients (*N* = 2; Temel et al., 2011; Nakano et al., 2018). Enzinger et al. (2020, 2021) recruited patients with colorectal and pancreatic cancer. Nipp et al. (2020) recruited patients with gastrointestinal and lung cancer. The other studies included patients with any type of primary cancer (Lippe et al., 2020; Chen et al., 2020; Sigler et al., 2022; Prigerson et al., 2023; Shen et al., 2024).

### Study quality

Using the CRBT and RoBANS, 10 of the 13 included studies were assessed as having a high risk of bias, with only three study being rated as having a low risk of bias. The quality assessment of each study is presented in Table 2, as detailed in Supplementary file 2.

The risk of bias in RCT studies primarily stemmed from the randomization process and the lack of blinding. Most studies were prone to the risk of selection bias due to inadequate randomization procedures and failure to ensure allocation concealment. Because the nature of psychological interventions, it was challenging to blind the study participants. The risk of bias in non-RCT studies usually caused by confounding factors in design and analysis not being accounted for.

### Prognostic understanding measures

Eleven studies assess the patient’s prognostic understanding using semi-structured interviews. Patients were typically asked several questions to assess their terminal illness acknowledgment, recognition of their incurable disease status, knowledge of the advanced stage of their disease, and expectation to live months as opposed to years. The patient’s responses were subsequently coded to assess whether the patient understands the prognosis and the level of their comprehension. Only two studies used assessment tools to evaluate prognostic understanding (Lippe et al., 2020; Nipp et al., 2020). Lippe et al. (2020) used the Prognosis and Treatment Perception questionnaire (PTPQ) to measure patients’ prognostic understanding. Nipp et al. (2020) used the Brief Illness Perceptions Questionnaire (BIPQ) to assess patients’ prognostic understanding.

### Intervention details

The characteristics of each intervention study are presented in Table 2. Interventions aimed at improving prognostic understanding in patients with advanced cancer included decision aids (DAs) - based interventions, prognostic discussions, advance care planning (ACP), and palliative care. These studies exhibit significant differences in the specific details of the interventions.

### Outcomes

#### Decision aids-based interventions

Five studies evaluated the impact of DAs - based interventions on the prognostic understanding among cancer patients, while the results of these five studies are inconsistent (Leighl et al., 2011; Nakano et al., 2018; Enzinger et al., 2020; AlSagheir et al., 2020; Enzinger et al., 2021). The DAs - based interventions in these studies were presented in the form of booklets, videos, audio recording, audiotape, or compact disk, which provided information including the goals of palliative care with or without chemotherapy, treatment options, treatment benefits, response rates, and estimated survival rates. Leighl et al. (2011) found support for DAs - based interventions in their RCT involving 207 patients, patients in the intervention group demonstrated a significant enhancement in their understanding of prognosis, with this effect becoming increasingly pronounced 1 to 2 weeks following the initial consultation (*p* < 0.001). A study of distributing educational brochures and videos to convey palliative chemotherapy information to patients with advanced colorectal and pancreatic cancer. The results showed that the correct understanding of the side effects of chemotherapy among patients in the intervention group was significantly higher than that in the control group, but the difference was not statistically significant (Enzinger et al., 2020). Besides, offering optional life expectancy information within the palliative chemotherapy education intervention had no overall effect on patients’ self-estimates of life expectancy, but patients in the pancreatic cancer subgroup developed more realistic prognostic expectations (*p* < 0.01), in the colorectal cancer subgroup, self-estimates of life expectancy tend to be more optimistic (Enzinger et al., 2021). DAs-based approaches did not significantly enhance patient prognostic understanding in individuals with metastatic colorectal cancer in Saudi Arabia (AlSagheir et al., 2020) or in those diagnosed with advanced non-small-cell lung cancer in Japan (Nakano et al., 2018).

### Prognostic discussions interventions

Four of the 13 studies evaluated the use of prognostic discussions interventions for patients with cancer. Communicating Oncologic Prognosis with Empathy (COPE) communication guide help promote a more consistent understanding of prognosis and treatment goals between oncologists and patients, patients’ understanding of prognosis after intervention was more accurate than before intervention (*p* < 0.001; Lippe et al., 2020). Step et al. (2019) evaluated the impact of team-based discussions on prognosis and treatment goals on breast cancer patients’ understanding of disease curability, results showed that a higher proportion of patients in the intervention group had a more accurate understanding of the curability of the disease (80.0 vs. 20.0%). Prigerson et al. (2023) developed the “Giving Information Simply and Transparently” (GIST) communication technique for oncologists, to help oncologists in using simple words and phrases to communicate prognosis with patients. Results indicated that GIST intervention is ineffective in improving prognostic understanding among patients with advanced cancer. Similarly, a communication intervention delivered remotely by licensed social workers also did not improve the prognostic understanding of patients with advanced cancer in the absence of a control group (Shen et al., 2024).

### Advance care planning interventions

Only one study has described ACP interventions aimed at improving the prognostic understanding in patients with advanced cancer. Chen et al. (2020) evaluated the effectiveness of individualized interactive ACP interventions on the prognostic understanding by assessing patients’ readiness for prognostic information and their needs for such information, it facilitated the transition of patients to accurate prognostic understanding and increased the time spent in the accurate prognostic understanding state. In comparison to the control group, patients in the experimental group exhibited significantly higher odds of accurate prognostic understanding during the three time periods of 61–90, 91–120, and 121–150 days before death, with adjusted odds ratios (95% confidence intervals) of 2.04 (1.16–3.61), 1.94 (1.09–3.45), and 1.93 (1.16–3.21), respectively. At present, there is insufficient information to make a conclusive statement regarding the effectiveness of ACP intervention in improving the prognostic understanding of patients with advanced cancer.

### Palliative care interventions

Three studies evaluated the impact of palliative care interventions on the prognostic understanding among cancer patients, led by geriatricians, oncology nurses, and palliative care team, respectively (Temel et al., 2011; Nipp et al., 2020; Sigler et al., 2022). For patients with metastatic non–small-cell lung cancer (NSCLC), the early palliative care intervention led by palliative care team had a large long-term effect on prognostic understanding. Results showed that a lower percentage of patients receiving early palliative care reported their cancer to be curable at 12 weeks (22.2 vs. 39.5%), and this difference persisted at 18 weeks (17.8 vs. 36.1%), but no significant differences were observed in patients’ perspectives on treatment goals between the study groups (Temel et al., 2011). However, neither palliative care interventions led by geriatricians (Nipp et al., 2020) nor those led by oncology nurses (Sigler et al., 2022) have shown an improvement in the understanding of prognosis.

## Discussion

This systematic literature review identified 13 studies, from six countries, that studied the effectiveness of interventions aimed at enhancing the prognostic understanding in patients with advanced cancer. Most studies were published within the past 5 years, with nine studies primarily aimed at enhancing prognostic understanding in patients with advanced cancer. This indicates that the importance of prognostic understanding among cancer patients may have gained broader recognition recently.

This review found that most studies focus on the feasibility results of small sample sizes (Nakano et al., 2018; Step et al., 2019; Lippe et al., 2020; Nipp et al., 2020; Prigerson et al., 2023; Shen et al., 2024), which represents the growing interest to collect preliminary feasibility data for interventions that focus on improving prognostic understanding in patients with advanced cancer.

A recent systematic review examined interventions evaluated within a randomized controlled design for their impact on prognostic understanding, finding that these interventions could improve prognostic understanding in patients with advanced stages of life-limiting illness (George et al., 2022). The interventions included providing information, offering decision and emotional support, assisting with advance care planning, using informational aids or communication training. However, it remains unclear which components of the interventions are the most important and effective, as no data have been presented to indicate which aspects of the interventions had the greatest impact. In this systematic review, we found that patients’ understanding of prognosis improved significantly when healthcare professionals directly engaged with patients in discussions about their prognosis (Temel et al., 2011; Leighl et al., 2011; Step et al., 2019; Lippe et al., 2020; Chen et al., 2020), as opposed to merely providing decision aids (AlSagheir et al., 2020; Enzinger et al., 2020; Enzinger et al., 2021). The effectiveness of such interventions may stem from the professional backgrounds of the interventionists in oncology and palliative care. Intervention work is typically conducted by specialized medical personnel, palliative care teams, or multidisciplinary teams, some studies have implemented specialized training in prognosis communication for interventionists. Studies have demonstrated that prognostic communication conducted by qualified professionals or multidisciplinary teams can facilitate the effective transmission of prognostic information (Cannone et al., 2019; Guo et al., 2025). Furthermore, these interventions are characterized by continuity, facilitated through multiple follow-up contacts. Lastly, the intervention process relies on a long-term doctor-patient care relationship, wherein structured conversations and decision aids are seamlessly integrated into daily communication rather than being isolated from the clinical environment. The Guidelines Committee of the European Society for Medical Oncology (ESMO) emphasized that patients place significant importance on the continuity of care services (Stiefel et al., 2024). For patients, continuity of care fosters a sense of security within the healthcare system and may enhance the development of a trusting relationship with their clinicians (Bazargan et al., 2021). The ESMO also noted that the effective implementation of communication depends on the subjective and situational nature of communication, which requires clinicians to flexibly apply their communication skills in specific clinical scenarios (Stiefel et al., 2024).

Interventions that relied on DAs were largely ineffective in improving prognostic understanding. Only a supporting treatment decision making intervention that used DAs (take-home booklet with audio recording, reviewed by an oncologist) to deliver difficult prognostic information to patients showed positive effect on prognostic understanding, besides, this effect became more pronounced 1 to 2 weeks after the initial consultation (Leighl et al., 2011). This implies that patients may not have fully understood their condition and the potential impacts of the treatment during the initial stages of consultation, potentially leading to inappropriate decision-making. Moreover, oncologists received training on the utilization of DAs during consultations, which they subsequently incorporated into their communication practices and revisited during follow-up visits (Leighl et al., 2011). Throughout the intervention, patients had the opportunity to communicate face-to-face with the same oncologist responsible for their treatment and follow-up. This allowed them to raise questions and receive professional answers, potentially enhancing the effectiveness of the intervention. Furthermore, the discussion of booklet content within an established therapeutic relationship may elucidate why patients’ understanding of prognosis improved more significantly within 1 to 2 weeks following the initial consultation. It is evident that patient education and the routine implementation of delayed decision-making are crucial in the metastatic setting, as they can significantly enhance informed decision-making by patients (Leighl et al., 2011).

DAs-based approaches did not enhance the prognostic understanding of patients with advanced cancer in non-Western countries, including Saudi Arabia (AlSagheir et al., 2020) and Japan (Nakano et al., 2018). This may be attributed to the fact that intervention programs developed in Western cultures often do not align with the expectations for prognostic information disclosure among non-Western populations. This discrepancy may arise from the latter’s comparatively lower emphasis on patients’ rights to independent medical decision-making (Tripodoro et al., 2024; Zhao et al., 2025; Martina et al., 2021).

Enzinger et al. (2021) found that in palliative chemotherapy education interventions, the provision of additional options to review survival statistics and disclose life expectancy information did not significantly affect patients’ self-assessment of their life expectancy. However, within the pancreatic cancer subgroup, patients randomized to the intervention group exhibited more realistic estimates of their life expectancy. Conversely, in the colorectal cancer subgroup, those randomized to the intervention group appeared to have less realistic estimates. Compared to patients with advanced pancreatic cancer, those with advanced colorectal cancer generally have a better prognosis, with up to 10–20% surviving beyond 5 years (Loupakis et al., 2014; Venook et al., 2017). These statistics may contribute to unrealistic expectations regarding life expectancy among patients with colorectal cancer. Furthermore, patients with colorectal cancer are more likely to encounter long-term survivors, whereas patients with pancreatic cancer are relatively rare (Suzuki et al., 2025; Hukkinen et al., 2025). This difference may influence patients’ perceptions of prognosis. Additionally, for tumors with extremely poor prognoses, such as pancreatic cancer, clinicians typically communicate prognosis in a more straightforward and clear manner (Blakely et al., 2017). In contrast, the prognosis for metastatic colorectal cancer is more uncertain, leading to more cautious communication from doctors (Liu et al., 2025). Therefore, the impact of DAs interventions on the prognostic understanding of patients with advanced cancer may be limited and could be influenced by factors such as the cultural background, type of cancer, patient’s social environment, and the clinician’s communication style.

Promoting patients’ accurate understanding of prognosis has a dual effect. On one hand, it aids patients in making informed end-of-life care decisions, reduces non-beneficial end-of-life treatments, and allows sufficient time for patients to plan important life events. On the other hand, clear communication of prognostic information may also induce negative psychological reactions in patients, such as despair and other forms of emotional distress (Meghani and Hinds, 2015; Gilligan et al., 2017). Communication about the prognosis of advanced cancer remains one of the most challenging discussions in healthcare. Physicians often worry that conveying bad news about diagnoses and prognoses to patients may diminish their hope (Fallowfield et al., 2002). Recommendations for discussing prognosis in advanced illness include planning and documenting the discussion, adapting to the patient’s preferences for the discussion, utilizing team-based discussions, and reiterating and discussing the patient’s preferences for aggressive care and end-of-life issues (Bradley et al., 2001; Eggly et al., 2006; Clayton et al., 2007; Wittenberg-Lyles et al., 2008; Walling et al., 2008; Saraiya et al., 2008; Finlay and Casarett, 2009; Mack et al., 2010; Zucca et al., 2017). The findings of Step et al. (2019) support the recommendations, as they discovered that, compared to the control group, patients with advanced breast cancer who underwent team-based discussions on prognosis and treatment goals had a more realistic understanding of their prognosis and treatment objectives. Lippe et al. (2020) found that the use of a Communicating Oncologic Prognosis with Empathy (COPE) communication guide appeared to facilitate more consistent understanding of prognosis and treatment goals between oncologists and patients. Incorporating planned and structured prognostic discussions in late-stage cancer treatment may enhance patients’ sense of control in highly threatening and uncertain situations.

American Society of Clinical Oncology (ASCO) recommends integrating palliative care into the standard oncological treatment for all patients diagnosed with cancer (Ferrell et al., 2017). Recent evidence from a systematic review indicates that primary palliative care approaches are associated with improved physical symptoms and quality of life in patients, without resulting in adverse survival outcomes (Ernecoff et al., 2020). However, the impact of palliative care interventions on the prognostic understanding of patients with advanced cancer is limited. Temel et al. (2011) found that patients with metastatic NSCLC who received early palliative care were more likely to retain or develop an accurate understanding of their disease over time, but there was still a lack of precise understanding regarding the goals of cancer treatment. There exists a significant contradiction between the understanding of prognosis among patients with advanced cancer and their treatment goals. This discrepancy may be influenced by various factors, including psychological and sociocultural elements. Driven by death anxiety and a sense of loss of control, cancer patients may be more inclined to adopt denial coping strategies, refusing to accept the reality of an incurable disease while deliberately amplifying their hopes for a cure (DeMartini et al., 2019; Finkelstein et al., 2021). Moreover, influenced by traditional cultural beliefs, the patients tends to hold the notion that “the ideal goal of any disease treatment is a cure,” making it challenging to accept the prospect of living with a chronic illness (Hasegawa et al., 2024). Besides, the Care management by Oncology Nurses to address supportive care needs (CONNECT) intervention for patients with advanced cancer (Sigler et al., 2022) may be limited as it was delivered by oncology nurses rather than oncologists and focused on assessing the patient’s coping and encouraged discussions with the oncologists rather than explicitly addressing the prognosis with the patients.

Prognosis disclosure recommendations suggest developing interventions to improve prognosis understanding based on the patient’s readiness to receive prognostic information (Jackson et al., 2013). The larger RCTs of individualized, interactive, and ACP intervention by Chen et al. (2020) contained some of the most promising results of the review, which explicitly assessed the participants’ readiness for prognostic information before providing the interventions and encouraged them to communicate with their physicians about prognosis. This study demonstrated a significant enhancement in the development of accurate prognostic understanding among cancer patients, as well as a prolonged duration of maintaining the accurate prognostic understanding status. The effectiveness of this intervention may stem from the interventionists’ continuous assessment of participants’ readiness for prognostic information, exploration of their needs for such information, and timely clarification of any misconceptions regarding prognosis. This process facilitates an accurate understanding of prognosis among participants.

The essence of ACP lies in its focus on building relationships through effective communication, which fosters a trusting connection between healthcare providers and patients (van der Plas et al., 2022; McMahan et al., 2024). It systematically clarifies the patient’s values, care preferences, social support networks, and the involvement of family members in medical discussions, as well as identifying the medical decision-making agent in the context of disability (Tilburgs et al., 2018). This approach aids patients in addressing uncertainties related to future care and facilitates independent consultation and communication (Nouri et al., 2022). Through ongoing dialog rooted in trust, discussions surrounding care goals, the clinical trajectory of the disease, and potential complications enable patients to gain a comprehensive understanding of their prognosis and disease progression.

In this review, we found that the evidence for the effectiveness of most interventions on prognostic understanding is very limited, but there is evidence that discussions between cancer patients and their physicians can lead to a better understanding of prognosis (George et al., 2022). This highlights the importance of prognostic communication between healthcare providers and patients with advanced cancer. In these discussions, the clinical trajectory of cancer, life expectancy, and treatment-related information (such as the purpose of treatment, side effects, and benefits) should be clearly communicated to the patient. A comprehensive understanding of their prognosis enables patients to participate more actively in important medical decisions and care planning (van der Velden et al., 2020).

In the previous review, George et al. (2022) included 12 studies, of which 11 were conducted with cancer patients. Consequently, their conclusions were predominantly derived from patients with advanced cancer, which parallels the study population in this systematic review. In this systematic review, we have updated the previous work by George et al. (2022). Our study encompasses randomized controlled trials and pre-post studies, extends the search period to 2025, and performs a more comprehensive analysis of various types of interventions.

There are several limitations of this systematic review. First, most studies did not employ scales to assess the multidimensional structure of prognostic understanding in patients with advanced cancer. In addition to the limited number of studies and insufficient available data to date, this has also prevented us from quantitatively synthesizing the research findings to determine the optimal intervention strategies for improving cancer patients’ understanding of their prognosis. Second, only included published English-language studies, potentially omitting some relevant research. Third, due to the wide variations in research methodologies and the small sample sizes, we were unable to conduct a meaningful meta-analysis. Fourth, despite a comprehensive literature search, some studies may still have been excluded. Fifth, the risk of bias assessment indicated that, due to methodological issues, most of the studies were classified as having a high risk of bias. Therefore, the findings should be interpreted with caution. Last, most studies included in this systematic review did not provide specific details regarding various types of interventions, such as implementation sites, duration, and intensity. Consequently, it is impossible to determine whether these intervention details affect the enhancement of prognostic understanding. Further randomized controlled trials involving prognostic discussions led by healthcare professionals with palliative care training and communication skills, preferably conducted within longitudinal consultations, are needed to confirm whether interventions grounded in high-quality communication and therapeutic relationships are associated with improved prognostic understanding.

## Conclusion

This systematic review aims to identify methods for developing prognosis understanding improvement programs for patients with advanced cancer by systematically reviewing the literature on prognosis understanding training programs for such patients. The methods used to assess prognostic understanding in the studies included in this review varied; nine studies considered prognostic understanding as the primary research outcome. Various prognostic understanding intervention studies have been conducted on patients with advanced cancer, serving as stepping stones for developing comprehensive prognostic understanding improvement programs. Future prospective randomized controlled studies are necessary to further validate whether interventions based on high-quality communication and therapeutic relationships can enhance prognostic understanding in patients with advanced cancer.

## Data Availability

The original contributions presented in the study are included in the article/Supplementary material, further inquiries can be directed to the corresponding author.
