# Supplementary material for: Prognostic understanding interventions in patients with advanced cancer: a systematic review
Source: Front Psychol. 2026 Jun 3;17:1824994. doi: 10.3389/fpsyg.2026.1824994 (PMC13272489; doi:10.3389/fpsyg.2026.1824994)
Supplement: Supplementary file 1 [file Table_1.DOCX]

| Supplementary-Table 1: Search strategies according to the database until February 2025 |
| --- |
| **Web of Science** |
| ***Participant (1)*** |
| TS=(Cancer OR tumor OR neoplasm OR carcinoma )  Limits: Articles; Topic |
| ***Primary outcome (2)*** |
| TS= (Prognostic understanding OR prognostic awareness OR prognostic perception OR illness expectation OR prognosis OR prognoses OR life expectancy)  Limits: Articles; Topic |
| ***Study design (3)*** |
| TS=(Randomized controlled trial OR controlled clinical trial OR randomization OR pretest posttest control group design OR pretest posttest design OR random*OR intervention)  Limits: Articles; Topic |
| In total, (1) AND (2) AND (3) |
| **PUBMED** |
| ***Participant (1)*** |
| Cancer OR tumor OR neoplasm OR carcinoma  Limits: Title/Abstract |
| ***Primary outcome (2)*** |
| Prognostic understanding OR prognostic awareness OR prognostic perception OR illness expectation OR prognosis OR prognoses OR life expectancy  Limits: Title/Abstract |
| ***Study design (3)*** |
| Randomized controlled trial OR controlled clinical trial OR randomization OR pretest posttest control group design OR pretest posttest design OR random* OR intervention  Limits: Title/Abstract |
| In total, (1) AND (2) AND (3) |
| **EMBASE** |
| ***Participant (1)*** |
| 'Cancer'/exp OR 'tumor'/exp OR 'neoplasm'/exp OR 'carcinoma'/exp |
| ***Primary outcome (2)*** |
| 'Prognostic understanding' OR 'prognostic awareness' OR 'prognostic perception' OR 'illness expectation' OR 'prognosis' OR ' prognoses ' OR ' life expectancy' |
| ***Study design (3)*** |
| 'Randomized controlled trial'/exp OR 'controlled clinical trial'/exp OR 'randomization'/exp OR 'pretest posttest control group design'/exp OR 'pretest posttest design'/exp OR 'intervention'/exp |
| In total, (1) AND (2) AND (3) |
| **SCOPUS** |
| ***Participant (1)*** |
| TITLE-ABS-KEY ( cancer OR tumor OR neoplasm OR carcinoma )  Limits: Title or abstract or keywords |
| ***Primary outcome (2)*** |
| TITLE-ABS-KEY ( "Prognostic understanding" OR "prognostic awareness" OR "prognostic perception" OR "illness expectation" OR " prognosis " OR " prognoses " OR " life expectancy " )  Limits: Title or abstract or keywords |
| ***Study design (3)*** |
| TITLE-ABS-KEY ( "Randomized controlled trial" OR "controlled clinical trial" OR randomization OR "pretest posttest control group design" OR "pretest posttest design" OR "random*" OR intervention )  Limits: Title or abstract or keywords |
| In total, (1) AND (2) AND (3) |
