# Supplementary material for: Prognostic understanding interventions in patients with advanced cancer: a systematic review
Source: Front Psychol. 2026 Jun 3;17:1824994. doi: 10.3389/fpsyg.2026.1824994 (PMC13272489; doi:10.3389/fpsyg.2026.1824994)
Supplement: Supplementary file 2 [file Table_2.DOCX]

Supplementary-Table 2: Summary of RCTs studies appraisal based on Cochrane Risk of Bias Tool

|  | D1:Bias arising from the randomization process. | D2: Bias due to deviations from intended intervention. | D3: Bias due to missing outcome data. | D4: Bias in measurement of the outcome. | D5: Bias in selection of the reported result. | Overall |
| --- | --- | --- | --- | --- | --- | --- |
| AlSagheir et al., (2020) | L | L | H | H | H | H |
| Enzinger et al., (2020) | H | H | L | H | L | H |
| Enzinger et al., (2021) | H | H | L | H | L | H |
| Leighl et al., (2011) | L | L | L | H | L | L |
| Chen et al., ( 2020) | H | L | H | H | L | H |
| Nipp et al., (2020) | H | L | L | L | L | L |
| Sigler et al., (2022) | H | L | H | H | L | H |
| Temel et al., (2011) | H | L | H | H | L | H |
| Step et al., (2019) | H | L | H | H | L | H |
| Prigerson et al., (2023) | H | L | H | H | L | H |

Supplementary-Table 3: Summary of Non-RCT studies appraisal based on Cochrane Risk of Bias Tool

|  | D1: Bias due to confunding. | D2: Bias due to selection of participants. | D3: Bias in classification of interventions. | D4: Bias due to deviations from intended intervention. | D5: Bias due to missing data. | D6:Bias in measurement of outcomes. | D7: Bias in selection of the reported result. | Overall |
| --- | --- | --- | --- | --- | --- | --- | --- | --- |
| Nakano et al., (2018) | H | H | H | H | L | H | L | H |
| Lippe et al., (2020) | H | H | L | L | L | L | L | L |
| Shen et al., (2024) | H | H | H | H | L | H | L | H |
